# Tree Fern *Cyathea lepifera* May Survive by Its Phytotoxic Property

**DOI:** 10.3390/plants9010046

**Published:** 2019-12-28

**Authors:** Noriyuki Ida, Arihiro Iwasaki, Toshiaki Teruya, Kiyotake Suenaga, Hisashi Kato-Noguchi

**Affiliations:** 1 Department of Applied Biological Science, Faculty of Agriculture, Kagawa University, Miki, Kagawa 761-0795, Japan; hisashi@ag.kagawa-u.ac.jp; 2 Department of Chemistry, Faculty of Science and Technology, Keio University, 3-14-1 Hiyoshi, Kohoku, Yokohama 223-8522, Japan; a.iwasaki@chem.keio.ac.jp (A.I.); suenaga@chem.keio.ac.jp (K.S.); 3 Faculty of Education, University of the Ryukyus, Nishihara, Okinawa 903-0213, Japan; t-teruya@edu.u-ryukyu.ac.jp

**Keywords:** *Cyathea lepifera*, tree fern, growth inhibitor, phytotoxicity

## Abstract

Cyatheaceae (tree ferns) appeared during the Jurassic period and some of the species still remain. Those species may have some morphological and/or physiological characteristics for survival. A tree fern was observed to suppress the growth of other ligneous plants in a tropical forest. It was assumed that the fern may release toxic substances into the forest floor, but those toxic substances have not yet been identified. Therefore, we investigated the phytotoxicity and phytotoxic substances of *Cyathea lepifera* (J. Sm. ex Hook.) Copel. An aqueous methanol extract of *C. lepifera* fronds inhibited the growth of roots and shoots of dicotyledonous garden cress (*Lepidum sativum* L.), lettuce (*Lactuca sativa* L.), and alfalfa (*Medicago sativa* L.), and monocotyledonous ryegrass (*Lolium multiflorum* Lam.), timothy (*Phleum pratense* L.), and barnyardgrass (*Echinochloa crus-galli* (L.) P. Beauv.). The results suggest that *C. lepifera* fronds may have phytotoxicity and contain some phytotoxic substances. The extract was purified through several chromatographic steps during which inhibitory activity was monitored, and *p*-coumaric acid and (-)-3-hydroxy-*β*-ionone were isolated. Those compounds showed phytotoxic activity and may contribute to the phytotoxic effects caused by the *C. lepifera* fronds. The fronds fall and accumulate on the forest floor through defoliation, and the compounds may be released into the forest soils through the decomposition process of the fronds. The phytotoxic activities of the compounds may be partly responsible for the fern’s survival.

## 1. Introduction

Cyatheaceae, a tree fern family, is distributed in tropical and subtropical forests. The tree ferns grow to 20 m high and have very large fronds reaching 3–4 m in length. Cyatheaceae appeared during the Jurassic period and are considered living fossils. Most species are extinct, but some have survived [1]. Those surviving species may have special morphological and/or physiological traits for survival.

The spatial occupation of *Cyathea muricata* (Cyatheaceae) in tropical forests was investigated in Guadeloupe in the Caribbean, and the tree fern was found to have suppressed the growth of other ligneous plants. This phenomenon was assumed to be caused by toxic substances released from the fern into the forest floor [2]. However, the substances have not yet been identified, and how those substances are released into the environment is also unknown.

*Cyathea lepifera* (Cyatheaceae) is distributed in the mountains of East and Southeast Asia and grows up to 15 m high. The canopy openness of the fern in the forests was 4-fold greater than two other closely related tree ferns, *C. spinulosa* and *C. podophylla.* The tree ferns drop their old fronds through defoliation and accumulate on the forest floor. The life span of *C. lepifera* fronds is 6–7 months, which is significantly shorter than the other two species [3]. Those observations suggest that more fallen fronds of *C. lepifera* accumulate on the forest floor than the other two species.

Several tree plants, such as walnut and pine, release toxic substances or allelopathic substances into the surrounding environment through fallen leaves [4,5,6]. Therefore, *C. lepifera* can be studied to determine if the tree ferns release toxic substances into the forest floor through defoliation of the fronds. The objective of this study was to investigate the phytotoxicity of *C. lepifera* fronds and identify the compounds that are phytotoxic active substances. 

## 2. Results 

### 2.1. Activity of the Extracts of C. lepifera Fronds 

**Figure 1:**
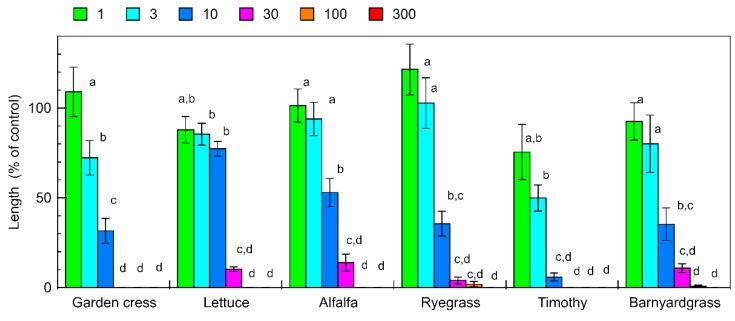
Effects of aqueous methanol extracts of *C. lepifera* fronds on the root growth of garden cress, lettuce, alfalfa, ryegrass, timothy, and barnyardgrass. Concentrations of the tested samples corresponded to the extract obtained from 1, 3, 10, 30, 100, and 300 mg dry weight of *C. lepifera* fronds per mL. Means ± SE from two independent experiments with 10 seedlings for each determination are shown. Different letters indicate significant differences (*p* < 0.05).

**Figure 2:**
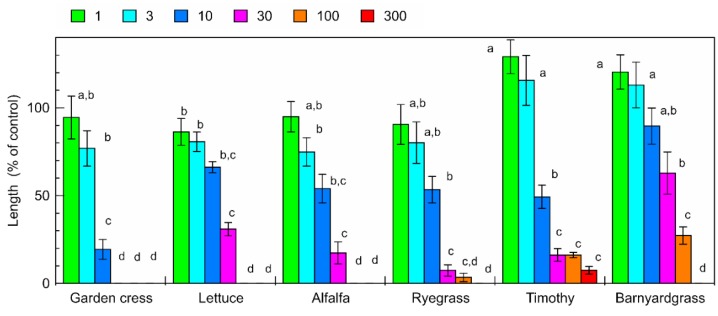
Effects of aqueous methanol extracts of *C. lepifera* fronds on the shoot growth of garden cress, lettuce, alfalfa, ryegrass, timothy, and barnyardgrass. Other details are as for Figure 1.

The aqueous methanol extracts of the *C. lepifera* fronds inhibited the root and shoot growth of garden cress, lettuce, alfalfa, ryegrass, timothy, and barnyardgrass (Figure 1 and Figure 2). The extract obtained from 30 mg of *C. lepifera* fronds, which was about 3 ppm in the bioassay solution, inhibited garden cress, lettuce, alfalfa, ryegrass, timothy, and barnyardgrass resulting in root growth of 0%, 10.3%, 13.9%, 4.0%, 0%, and 10.8% compared with that of control root growth, respectively, and inhibited garden cress, lettuce, alfalfa, ryegrass, timothy, and barnyardgrass resulting in shoot growth of 0%, 30.9%, 17.4%, 7.4%, 16.2%, and 62.9% compared with that of control shoot growth, respectively. Comparing the concentrations required for 50% growth inhibition (IC_50_ value), garden cress shoots were the most sensitive and barnyardgass shoots were the least sensitive to the extracts (Table 1).

### 2.2. Isolation of Active Substances in the C. lepifera Fronds 

**Figure 3:**
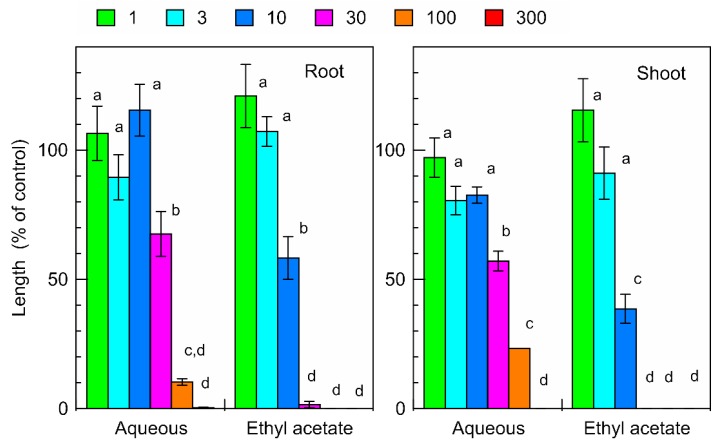
Effects of the ethyl acetate and aqueous fractions obtained from the extracts of *C. lepifera* fronds on the root and shoot growth of garden cress. Other details are as for Figure 1.

The extract of *C. lepifera* fronds was separated into ethyl acetate and aqueous fractions. Both fractions suppressed the root and shoot growth of the garden cress seedlings (Figure 3). The ethyl acetate and aqueous fractions obtained from 30 mg of *C. lepifera* fronds inhibited garden cress resulting in root growth of 1.6% and 67.6% compared with that of control root growth, respectively, and in shoot growth of 0% and 57.1% compared with that of control shoot growth, respectively. 

**Figure 4:**
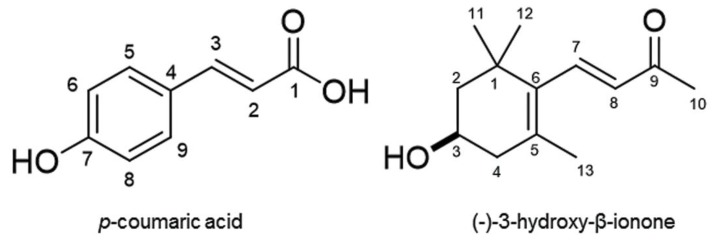
Chemical structures of *p*-coumaric acid and (-)-3-hydroxy-*β*-ionone.

The ethyl acetate fraction was then separated using a silica gel column, and the biological activity of all fractions was determined using a garden cress bioassay. Inhibitory activity was found in fractions 6 and 7. Fraction 6 was purified through chromatographic runs and the most active fraction was further purified during all the isolation processes. An active compound 1 (215 mg) was finally isolated by HPLC. Compound 1 has a molecular formula of C_9_H_8_O_3_ as suggested by HRESIMS *m*/*z* 163.0387 [M−H]^−^ (calcd for C_9_H_7_O_3_, 163.0395, Δ = −0.8 mmu); ^1^H NMR (400 MHz, CD_3_OD) δ_H_ 7.60 (d, *J* = 16.3 Hz, 1H, H-3), 7.45 (d, *J* = 8.9 Hz, 2H, H-5/9), 6.80 (d, *J* = 8.9 Hz, 2H, H-6/8), 6.28 (d, *J* = 16.4 Hz, 2H, H-6/8). Compound 1 was identified as *p*-coumaric acid (MW 164, Figure 4) by comparing its spectral data with previously reported data in the literature [7,8].

Fraction 7 of a silica gel column was also purified through chromatographic runs. The most active fraction was further purified and an active compound 2 (1.4 mg) was isolated by HPLC. Compound 2 has a molecular formula of C_13_H_20_O_2_ as suggested by HRESIMS *m/z* 209.1534 [M+H]^+^ (calcd for C_13_H_21_O_2_ 209.1542, Δ = −0.8 mmu); ^1^H NMR (400 MHz, CDCl_3_) δ_H_ 7.21 (d, *J* = 16.9 Hz, 1H, H-7), 6.11 (d, *J* = 16.9 Hz, 1H, H-8), 4.01 (m, 1H, H-3), 2.43 (dd, *J* = 17.2, 5.4 Hz, 1H, H-4), 2.30 (s, 3H, H-13), 2.08 (dd, *J* = 17.2, 9.7 Hz, 1H, H-4), 1.79 (m, 1H, H-2), 1.49 (m, 1H, H-2), 1.12 (s, 3H. H-11), 1.11 (s, 3H, H-12). The optical rotation of the compound was [α]_D_^26^ = −29.5 (*c* = 0.04, CHCl_3_). Compound 2 was identified as (-)-3-hydroxy-β-ionone (MW 208, Figure 4) by comparing its spectrum data with published data [9,10].

### 2.3. Inhibitory Activity of the Isolated Compounds

**Figure 5:**
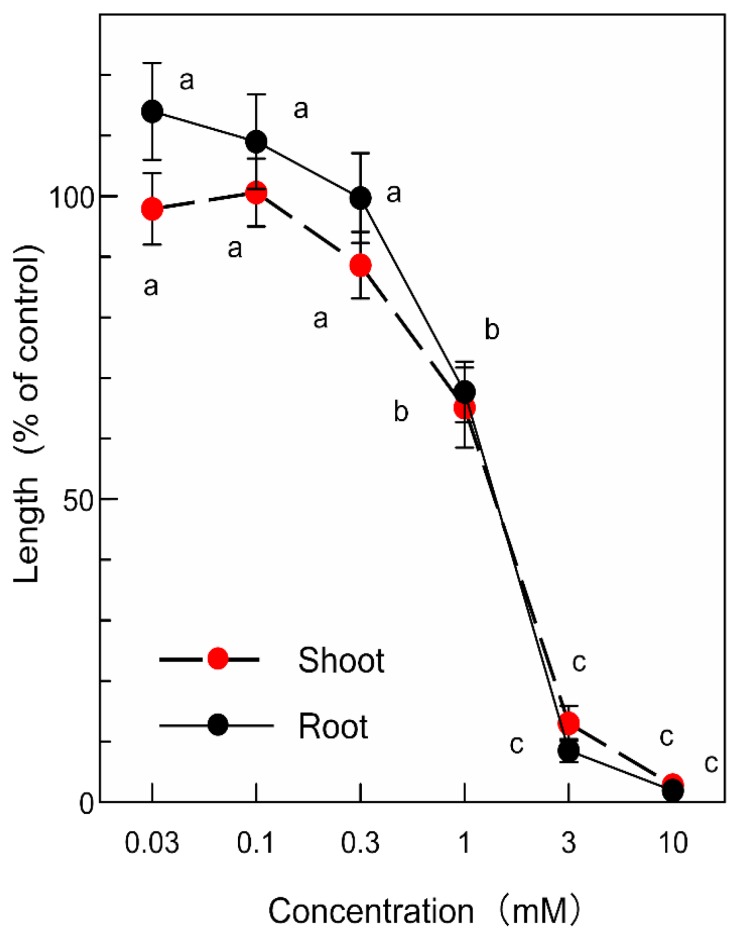
Effects of *p*-coumaric acid on the root and shoot growth of garden cress. Means ± SE from two independent experiments with 10 seedlings for each determination are shown. Different letters indicate significant differences (*p* < 0.05).

**Figure 6:**
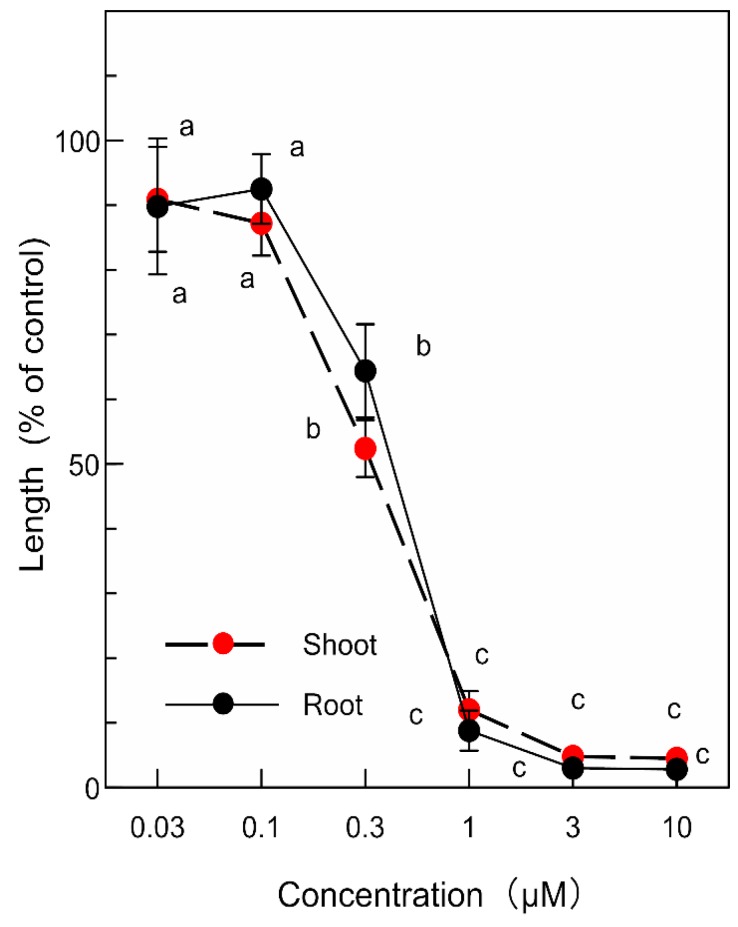
Effects of (-)-3-hydroxy-*β*-ionone on the root and shoot growth of garden cress. Other details are as for Figure 5.

*p*-Coumaric acid and (-)-3-hydroxy-*β*-ionone inhibited the growth of the garden cress roots and shoots at concentrations greater than 1 mM and 0.3 µM, respectively (Figure 5 and Figure 6). The inhibitory activity of the compounds increased with increasing concentrations of the compounds. Comparing IC_50_ values, the inhibitory activity of (-)-3-hydroxy-*β*-ionone was 100-fold greater than that of *p*-coumaric acid (Table 2). 

## 3. Discussion

The aqueous methanol extracts of the *C. lepifera* fronds had an inhibitory effect on both the dicotyledonous plants (garden cress, lettuce, and alfalfa) and monocotyledonous plants (ryegrass, timothy, and barnyardgrass) in an extract-concentration-dependent manner (Figure 1 and Figure 2). However, the dicotyledonous plants had similar IC_50_ values for their roots and shoots, but the IC_50_ values of the roots of the monocotyledonous plants were 1.4 to 6.6 times greater than those of the shoots (Table 1). Therefore, the extract may have different actions against the dicotyledonous and monocotyledonous plants. The sensitivity of the garden cress roots and shoots was the highest among those of the test plants. Therefore, garden cress was selected as the test plant for the isolation of the phytotoxic active substances. Those results suggest that *C. lepifera* fronds may possess phytotoxicity and contain phytotoxic active substances.

The extract of *C. lepifera* fronds was separated into ethyl acetate and aqueous fractions and the inhibitory activity of the ethyl acetate fraction was greater than that of the aqueous fraction (Figure 3). Thus, isolation of phytotoxic active substances proceeded using the ethyl acetate fraction. Compound 1 was isolated from fraction 6 of the silica gel column chromatography and identified as *p*-coumaric acid (Figure 4) by comparing its spectral data with previously reported data in the literature [7,8]. *p*-Coumaric acid, a hydroxycinnamic acid, has been found in several plant species [5,11]. Compound 2 was isolated from fraction 7 of the silica gel column and identified as (-)-3-hydroxy-β-ionone by comparing its spectrum data with published data [9,10]. 3-Hydroxy-β-ionone has also been isolated from other plants including moss species [12,13,14]. 

We isolated 215 mg of *p*-coumaric acid and 1.4 mg of (-)-3-hydroxy-β-ionone from 1.5 kg *C. lepifera* fronds as described above. Thus, the concentration of *p*-coumaric acid in *C. lepifera* fronds is probably 150-fold greater than that of (-)-3-hydroxy-β-ionone. However, the inhibitory activity of (-)-3-hydroxy-β-ionone may be 100-fold greater than that of *p*-coumaric acid because of IC_50_ values (Table 2). Therefore, those compounds may contribute equally to the growth inhibitory effect of the extract of *C. lepifera* fronds. 

*p*-Coumaric acid is an intermediate in the phenylpropanoid pathway and a precursor of the biosynthesis of flavonoids and monolignols [15]. *p*-Coumaric acid has been found in several plant root exudates [16,17] and in soils cultivated with crops [18,19]. It has also identified in decomposing plant residues [20,21] and in soils after being incorporated with plant materials [22,23]. Those observations indicate *p*-coumaric acid was liberated into the soils during the decomposition of the plants. *p*-Coumaric acid was reported to have growth inhibitory activity against a wide range of plant species. Based on the findings of the existence of *p*-coumaric acid in soils and its growth inhibitory activity, *p*-coumaric acid is considered to act as an allelopathic substance [5,9,21]. It was also found that *p*-coumaric acid induces monolignol polymerization and solidifies soybean root cell walls, resulting in inhibition of root growth [24]. A strong accumulation of *p*-coumaric acid occurred after application of the herbicides chloresulfuron and imazethapyr, which inhibited acetolactate synthase, and some of the physiological effects caused by the herbicides closely resembled those caused by *p*-coumaric acid [25]. 

(-)-3-Hydroxy-*β*-ionone, a norisoprenoid, is a cleavage product of zeaxanthin and is present during fruit development [13,26,27]. It has also been shown to accumulate in etiolated bean seedlings through light irradiation, resulting in light-induced growth inhibition of the seedlings [28]. (-)-3-Hydroxy-*β*-ionone has also been isolated from several plant species and has growth inhibitory activity against other plant species [29,30,31]. The moss *Rhynchostegium pallidifolium* is allelopathic and its main allelopathic substance is (-)-3-hydroxy-*β*-ionone [14]. (-)-3-Hydroxy-*β*-ionone was also found in the growth medium of *R. pallidifolium* [32]. Based on the inhibitory activity and levels in the medium, (-)-3-hydroxy-*β*-ionone is able to cause 46%–64% of the observed allelopathic activity of *R. pallidifolium*. Thus, (-)-3-hydroxy-*β*-ionone possibly works as an allelopathic agent. 

Plants secrete many kinds of compounds from their roots, such as by proton-pumping and endoplasmic-derived exudation [33,34]. Many compounds are also liberated into the soils during decomposition processes of plant residues [21,35]. *C. lepifera* drops its old fronds, which accumulate on the forest floor. The life span of *C. lepifera* fronds is 6–7 months [3]. The fronds contain *p*-coumaric acid and (-)-3-hydroxy-*β*-ionone, which have growth inhibitory activity as described above. The compounds may also be released into forest soils through the decomposition process of *C. lepifera* fronds or secretion via roots or both. Therefore, those compounds may contribute to the phytotoxicity of *C. lepifera* and may be partly responsible for its survival. (-)-3-Hydroxy-*β*-ionone and *p*-coumaric acid were reported to have inhibitory activity against a wide range of plant species [5,9,21,29,30,31]. However, it is necessary to determine the inhibitory activity of those compounds on the plants species under tree fern *C. lepifera.*

## 4. Materials and Methods 

### 4.1. Plant Materials

Fronds of *Cyathea lepifera* (J. Sm. ex Hook.) Copel. were collected in Nishihara, Okinawa in 2015. Dicotyledonous garden cress (*Lepidum sativum* L.), lettuce (*Lactuca sativa* L.), and alfalfa (*Medicago sativa* L.), and monocotyledonous ryegrass (*Lolium multiflorum* Lam.), timothy (*Phleum pratense* L.), and barnyardgrass (*Echinochloa crus-galli* (L.) P. Beauv.) were chosen as test plants as described by [36,37,38]. 

### 4.2. Extraction and Bioassay

Fronds of *C. lepifera* (100 g dry weight) were cut into small pieces and extracted with 2.5 L of 70% (*v*/*v*) aqueous methanol for two days. After filtration using filter paper (No. 2; Toyo, Tokyo, Japan), the residue was extracted again with 2 L of methanol for two days and filtered, and the two filtrates were combined. 

An aliquot of the extract (final assay concentration of tested samples corresponded to the extract obtained from 1, 3, 10, 30, 100, and 300 mg dry weight of *C. lepifera* fronds per mL) was evaporated to dryness, dissolved in 0.2 mL of methanol, and added to a sheet of filter paper (No. 2) in a 3 cm Petri dish. Methanol was evaporated in a fume hood. The filter paper in the Petri dishes was moistened with 0.8 mL of a 0.05% (*v*/*v*) aqueous solution of Tween 20. Ten seeds each of garden cress, lettuce, and alfalfa were then sown on the Petri dishes, and 10 seedlings each of timothy, ryegrass, and barnyardgrass were placed into the Petri dishes after germination in the dark at 25 °C for 36–48 h. The length of the roots and shoots of these seedlings was measured after 48 h of incubation in darkness at 25 °C, and the percentage length of the seedlings was determined by reference to the length of control seedlings. For control treatments, methanol (0.2 mL) was added to a sheet of filter paper in a Petri dish and evaporated as described above. Control seeds or seedlings were then placed onto the filter paper moistened with an aqueous solution of Tween 20. The bioassay was repeated two times using a randomized design with 10 plants for each determination. IC_50_ values on the test plant roots and shoots were determined using a logistic regression function based on the bioassay. 

### 4.3. Separation of the C. lepifera Frond Extract

*C. lepifera* fronds (1.5 kg dry weight) were extracted with 15 L of 70% (*v*/*v*) aqueous methanol and 15 L of methanol as described above, and concentrated at 40 °C in vacuo to produce an aqueous residue. The aqueous residue was then adjusted to pH 7.0 with 1 M phosphate buffer, partitioned three times against an equal volume of ethyl acetate, and separated into ethyl acetate and aqueous fractions. The biological activity of the two fractions was determined using a garden cress bioassay as described above.

The ethyl acetate fraction was then evaporated to dryness and separated on a column of silica gel (100 g, silica gel 60, 70–230 mesh; Merck), and eluted with 20%, 30%, 40%, 50%, 60%, 70%, and 80% ethyl acetate in *n*-hexane (*v*/*v*; 100 mL per step), ethyl acetate (100 mL), and methanol (200 mL). The biological activity of all separated fractions was determined using a garden cress bioassay. Two growth inhibitory active fractions were obtained by elution with 70% (fraction 6) and 80% (fraction 7) ethyl acetate in *n*-hexane. 

### 4.4. Purification of the Active Compound in Fraction 6 

Active fraction 6 obtained from the silica gel column was evaporated, and the residue was purified using a column of Sephadex LH-20 (100 g; GE Healthcare, Uppsala, Sweden) and eluted with 20%, 30%, 40%, 50%, 60%, 70%, 80%, and 90% (*v*/*v*) aqueous methanol and methanol (200 mL per step). The active fractions were eluted with 30% and 40% aqueous methanol. The two fractions were combined and evaporated to dryness. The residue was dissolved in 20% (*v*/*v*) aqueous methanol (2 mL), loaded onto an ODS cartridge (YMC-Dispo Pack AT ODS-25; YMC Ltd., Kyoto, Japan), and eluted with 20%, 30%, 40%, 50%, 60%, 70%, 80%, and 90% (*v*/*v*) aqueous methanol (120 mL per step) and methanol (240 mL). The active fraction was eluted with 40% aqueous methanol and evaporated to dryness. The residue was finally purified using reverse-phase HPLC (SPC-10A; Shimadzu, Kyoto; column, ODS AQ-325, 10 mm i.d. × 50 cm; YMC Ltd.) and eluted at a flow rate of 1.5 mL min^−1^ with 35% aqueous methanol and detected at 220 nm. Inhibitory activity was found in a peak fraction eluted in 96–98 min, yielding active compound 1. The compound was characterized using high-resolution ESI mass, H-NMR spectra (400 MHz, TMS as internal standard).

### 4.5. Purification of the Active Compound in Fraction 7 

Active fraction 7 obtained from the silica gel column was evaporated, and the residue was purified using a column of Sephadex LH-20 as described above. The active fraction was eluted with 40% aqueous methanol and evaporated to dryness. The residue was dissolved in 20% (*v*/*v*) aqueous methanol (2 mL) and loaded onto reverse-phase C_18_ cartridges (YMC-Dispo SPE ODS; YMC Ltd. Kyoto, Japan). The cartridges were eluted with 20%, 30%, 40%, 50%, 60%, 70%, 80%, and 90% (*v*/*v*) aqueous methanol and methanol (30 mL per step). The active fraction was eluted with 40% aqueous methanol and evaporated to dryness. The residue was finally purified using reverse-phase HPLC eluted at a flow rate of 1.5 mL min^−1^ with 45% aqueous methanol. Inhibitory activity was found in a peak fraction eluted in 135–137 min, yielding active compound 2. The compound was characterized using high-resolution ESI mass, H-NMR spectra (400 MHz, TMS as internal standard) and optical rotation.

### 4.6. Bioassay of the Isolated Compounds

The isolated compounds were separately dissolved in 0.2 mL of methanol and added to a sheet of filter paper (No. 2) in a 3 cm Petri dish. After methanol was evaporated, the Petri dishes was moistened with 0.8 mL of a 0.05% (*v*/*v*) aqueous solution of Tween 20. Final assay concentrations (0.03, 0.1, 0.3, 1, 3, 10 µM) of (-)-3-hydroxy-*β*-ionone were selected because of the activity of the compounds and those (0.03, 0.1, 0.3, 1, 3, 10 mM) of *p*-coumaric acid were selected. The biological activity was examined using a garden cress bioassay as described above.

### 4.7. Statistical Analysis

The bioassay was repeated two times using a completely randomized design with 10 plants for each determination. Significant differences among treatments within each test plant were examined using Duncan’s multiple comparison test at *p* < 0.05. 

## 5. Conclusions

An aqueous methanol extract of *C. lepifera* fronds inhibited the root and shoot growth of six test plants. The extract was then purified and *p*-coumaric acid and (-)-3-hydroxy-*β*-ionone were isolated. Those compounds may contribute to the inhibitory effects of *C. lepifera* fronds and be released into the forest floor through defoliation of its old fronds. The inhibitory activities of the compounds may be responsible for the phytotoxicity of *C. lepifera*, which may be involved in the fern’s survival.

## Figures and Tables

**Table 1:** The concentrations of the extracts of *C. lepifera* fronds required for 50% growth inhibition (IC_50_; g dry weight equivalent extract per mL) on the root and shoot growth of the test plants.

	Root	Shoot
Garden cress	5.80 ^a^	5.72 ^a^
Lettuce	15.4 ^c^	16.8 ^c^
Alfalfa	10.7 ^b^	11.3 ^b^
Ryegrass	7.72 ^a,^^b^	10.9 ^b^
Timothy	6.47 ^a,b^	12.3 ^b^
Barnyardgrass	6.74 ^a,b^	44.8

Different letters within the same column indicate significant differences (*p* < 0.05).

**Table 2:** IC**_50_** values of *p*-coumaric acid and (-)-3-hydroxy-*β*-ionone (µM) on the root and shoot growth of garden cress.

	Root	Shoot
*p*-Coumaric acid	1240 ^b^	1120 ^b^
(-)-3-Hydroxy-*β*-ionone	11.2 ^a^	10.7 ^a^

Different letters indicate significant differences (*p* < 0.05).
